# Three-dimensional assessment of facial asymmetry in Class III subjects. Part 1: a retrospective study evaluating postsurgical outcomes

**DOI:** 10.1007/s00784-022-04463-4

**Published:** 2022-03-23

**Authors:** Deepal Haresh Ajmera, Richard Tai-Chiu Hsung, Pradeep Singh, Natalie Sui Miu Wong, Andy Wai Kan Yeung, Walter Yu Hang Lam, Balvinder S. Khambay, Yiu Yan Leung, Min Gu

**Affiliations:** 1 Discipline of Orthodontics, Faculty of Dentistry, the University of Hong Kong, Hong Kong SAR, China; 2 Department of Computer Science, Chu Hai College of Higher Education, Hong Kong SAR, China; 3 Discipline of Oral and Maxillofacial Surgery, Faculty of Dentistry, the University of Hong Kong, Hong Kong SAR, China; 4 Department of Applied Oral Sciences & Community Dental Care, Faculty of Dentistry, the University of Hong Kong, Hong Kong SAR, China; 5 Discipline of Prosthodontics, Faculty of Dentistry, the University of Hong Kong, Hong Kong SAR, China; 6 Institute of Clinical Sciences, College of Medical and Dental Sciences, The School of Dentistry, University of Birmingham, Birmingham, UK

**Keywords:** Facial asymmetry, Maxillomandibular asymmetry 3D, Three-dimensional, Orthognathic surgery

## Abstract

**Objective:**

The present study aimed to determine the site and severity of maxillomandibular asymmetry before and after orthognathic surgery in asymmetric patients.

**Materials and methods:**

Presurgery and postsurgery cone beam computed tomography (CBCT) data of 21 facial asymmetry patients (7 males and 14 females, mean age: 23.0 ± 3.36 years) with soft tissue chin deviation ≥ 3 mm who had undergone bimaxillary surgery were evaluated. Seven midline and twenty bilateral hard tissue landmarks were identified for the evaluation of facial asymmetry and outcomes were assessed against age- and gender-matched control subjects.

**Results:**

In the asymmetry group, bilateral landmarks exhibited significant deviation in the mandible and midface regions. Before surgery, asymmetry was more severe at the mandibular midline and sites close to it, in the asymmetry group. Bimaxillary surgery proved to be highly effective, with a significant correction of the menton to a clinically normal value (2.90 mm, *p* < 0.001). After surgery, significant residual asymmetry was observed at the mental foramen (*p* = 0.001) in the R-L direction. Moreover, significant asymmetry persisted at the sigmoid notch (*p* = 0.001) in the S-I direction.

**Conclusions:**

Mandibular midline landmarks and chin peripheral regions contribute significantly to overall facial asymmetry characteristics. Despite significant correction after bimaxillary surgery, asymmetry persisted at several sites, thereby requiring secondary correction. Comprehensive 3D presurgical planning is central for asymmetry correction in a single surgery.

**Clinical relevance:**

The present study specifies the location of residual asymmetry sites and advocates the correction of those sites during initial surgery.

## Introduction

Aberration from absolute symmetry can be considered asymmetry, and the human face is not an exception to this state, since growth and developmental disparities may bring about some degree of facial asymmetry [1, 2]. Previous studies have associated facial asymmetry with a congenital or developmental disorder, as a feature of anarchic growth from environmental causes, or as a consequence of trauma, surgery, or disease [1, 3]. Facial asymmetry is a normal biological phenomenon, and the two halves of the face may not always be symmetric across the facial midsagittal plane. Any divergence or asymmetry beyond normal limits is cognitively detectable [4]. A discrepancy in facial architecture may not only affect normal oral functions and facial aesthetics but may also impact the person psychosocially [5, 6]. The incidence of clinically noticeable facial asymmetry among patients with dentofacial deformities has been reported to be 34–38.6% [7] compared with 23% in the orthodontic population [8]. In addition, the lower third of the face is the most frequent site affected by facial asymmetry, accounting for approximately 40–80% of cases [7, 9]. Excessive mandibular growth in prognathic mandible patients may contribute to this high prevalence [10].

Masuoka et al. suggested that clinically symmetric or mildly asymmetric patients might display severe skeletal asymmetry when diagnosed comprehensively [11]. A slight level of asymmetry is inherent to any normal human face and is tolerated within normal parameters; nevertheless, surgical correction is requisite for severely asymmetric faces associated with skeletal deformities [12, 13]. Hence, a precise, objective, and quantifiable assessment of the degree of asymmetry is indispensable for the diagnosis and treatment planning of facial asymmetry. Conventional techniques for the quantification of facial features are based on direct anthropometry and digital photography [12]; however, in the current era of digital imaging, several three-dimensional (3D) techniques have been utilized, including computed tomography (CT), cone beam computed tomography (CBCT), magnetic resonance imaging (MRI), stereophotogrammetry, and laser surface scanning, for the accurate assessment of asymmetry [14–16]. With the advent of advanced 3D software packages, it is possible to capture precise facial forms, and 3D reconstruction and facial disproportion measurements can also be performed with a high degree of accuracy [5, 16–18]. In addition, CBCT, when combined with recent 3D imaging tools, facilitates the registration of pre- and postsurgical radiographs with fewer magnification and distortion errors [19].

Hard tissues are important for CBCT registration and construction of a 3D coordinate system because they are more consistent and stable and such landmarks are more easily reproducible than those in soft tissues [20]. Furthermore, hard tissues are clearly visible in CBCT images. Accordingly, analyzing hard tissues while diagnosing facial asymmetry is central to desired treatment outcomes. Hard tissue analysis is a precondition for the preoperative simulation of surgical procedures and the evaluation of treatment results in facial deformity patients; however, previous studies have relied only on a few selected landmarks that do not represent true 3D surface morphology. Several studies on facial asymmetry have analyzed the outcome of orthognathic surgery (OGS) on soft tissues [21–23], and literature based on hard tissues is very rare [24]. Given that the comparison of postsurgical outcomes with normal controls is significant, as a socially acceptable postsurgical facial appearance is contingent upon the elective surgical procedure, the lack of a normal reference group in these studies prevents an unprejudiced evaluation of whether the outcome is ideal. In addition, hard tissue changes following OGS in patients with facial asymmetry have never been methodically studied for three-dimensional outcome measures (R, A, S; right-left, anterior–posterior, and superior-inferior). With this intent, we hypothesize that maxillomandibular asymmetry after surgery is significantly different from that presurgically and that the outcome measures post surgery are comparable to those for controls. Therefore, the present study aimed to determine the site and severity of maxillomandibular asymmetry before and after orthognathic surgery in asymmetric patients compared to normal controls. The primary outcome of the study was to evaluate the postsurgical changes in the asymmetry group and to ascertain the site and severity of any residual maxillomandibular asymmetry following surgical correction. This information will provide a greater understanding of surgical correction outcomes in three dimensions that may have clinical implications in modifying treatment plans and surgical approaches for enhanced aesthetics.

## Materials and methods

Ethics approval was obtained from the local institutional review board (IRB) of the University/Hospital Authority (approval number UW 19–377) before the commencement of this study.

### Study design

#### Asymmetry group

The sample for the asymmetry group consisted of 27 patients who had undergone orthognathic surgery between April 2012 and July 2019 at the Prince Philip Dental Hospital, University of Hong Kong. All patients fulfilled the following inclusion criteria: (1) had clinically corrected maxillomandibular asymmetry, i.e., soft tissue chin deviation less than 3 mm after surgery; (2) underwent bimaxillary surgery with no genioplasty, (3) were aged 18 to 40 years, (4) had a presurgical CBCT scan (*T*_0_) and an at least 6-month postsurgery CBCT scan (*T*_1_), (5) had no history of temporomandibular joint disorder, (6) had no history of craniofacial surgery or craniofacial syndromes, (6) had no clinically diagnosed orbital dystopia, and (7) had no diagnosis of hemifacial microsomia. The baseline characteristics of the study subjects are presented in Table 1.

**Table 1:** Patient characteristics in the asymmetry and control groups

Group	Sex		Total (*n*)	Age (years)	Surgery			Me deviation (mm)
	Male (*n*)	Female (*n*)		Mean ± SD	Le Fort I + BVSO^a^	Le Fort I + BSSO^b^	Le Fort I + VSO + SSO	Mean ± SD
Asymmetry group	7	14	21	23.0 ± 3.4	13	6	2	7.31 ± 4.10
Control group	7	14	21	23.0 ± 3.3	-	-	-	1.22 ± 0.80

^a^*BVSO*, bilateral vertical subsigmoid osteotomy

^b^*BSSO*, bilateral sagittal split osteotomy

Maxillary surgery was performed as either a one/two/or four piece LeFort I osteotomy for midfacial correction, while mandibular surgery was via bilateral vertical subsigmoid osteotomy (BVSO), or bilateral sagittal split osteotomy (BSSO). The surgeries were planned digitally and the surgical movements were executed according to the surgical plan. Titanium mini-plates and screws were utilized for semi-rigid fixation. Postoperatively, all the patients were instructed to undertake active physiotherapy including mouth opening and lateral excursion of jaw movements for 2 weeks. Following physiotherapy, postsurgical orthodontic treatment was initiated and implemented for a period of 6 months to 1 year.

#### Control group

The control group consisted of age- and sex-matched subjects selected from a pool of patients who had CBCT scans taken in 2015 from the same hospital for nonorthognathic surgery reasons (e.g., implants or complex extractions). Apart from age and sex, additional inclusion criteria were as follows: (1) no clinically apparent maxillomandibular asymmetry (soft tissue chin deviation < 3 mm); (2) class I skeletal pattern; (3) well-aligned dental arches; (4) no posterior dental crossbite; (5) no history of temporomandibular disorder; and (5) no history of craniofacial surgery or craniofacial syndromes (Table 1).

#### Sample size calculation

Sample size calculation was based on the consideration of detecting a clinically relevant mean difference of at least 0.66 mm (standard deviation of 0.5 mm) in the measurements after surgery [20]. Accordingly, with an alpha level of 0.05, study power of 0.95, and an effect size of 1.32, a minimum sample size of 32 (16 in each group) was calculated using G*Power (version 3.1.9.2, Kiel University, Germany) [25].

### Data collection

#### CBCT acquisition

CBCT scans of the maxillofacial region were obtained using a ProMax 3D Mid (Planmeca, Helsinki, Finland) using the following parameters: 90 kVp, 400 μm voxel size, 4.7 s scan time, and 20 cm × 17 cm field of view. CBCT scanning was performed with the patients sitting comfortably, and their head position, such as the Frankfurt horizontal (FH) plane, was parallel to the floor. Throughout the scanning procedure, patients were instructed to maintain light contact of their teeth with the bite-peg, and the lips and labiomental soft tissues at rest, to eliminate possible CR-CO (centric relation-centric occlusion) discrepancy and overclosure of the mandible which is very common in class III cases. The CBCT scans were stored in Digital Imaging and Communications in Medicine (DICOM) format and then transferred to 3D Slicer 4.10, an open-source medical image processing software platform (www.slicer.org) for analysis [26].

#### Orientation of the CBCT volume

**Fig. 1:**
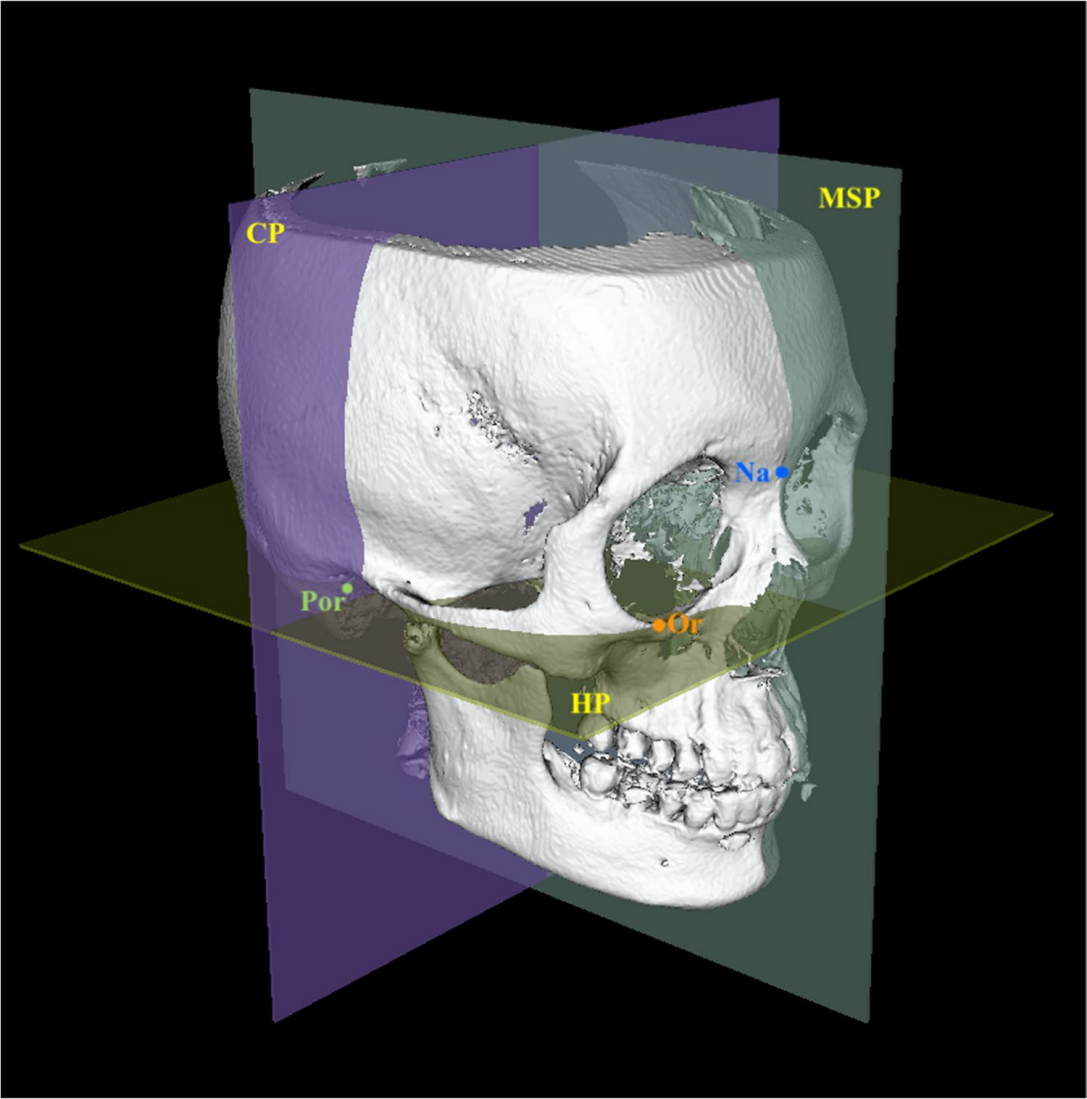
3D view of the reference planes used. HP, horizontal plane (green)—passing through the orbitales (Or) and porion (Por). MSP, midsagittal plane (blue)—plane passing through nasion (Na) and sella (S), and perpendicular to HP. CP, coronal plane (purple)—plane passing through porion (Por) and perpendicular to the HP and MSP

This involved three steps: the first was to convert the DICOM data into surface data, the second was to manually landmark the 3D images, and the third was to reorient the 3D images into a standardized position. For each subject, a 3D rendered surface model was generated from the CBCT volume using Slicer software. The threshold value was adjusted between 250 and 320 HU (Hounsfield units), followed by bone segmentation using the “Editor tool.” After manual digitization of landmarks using the “Markups tool” in the Slicer software, 3D reference planes were established, which were defined as follows: the plane passing through the right and left orbitales and the left porion point was defined as the horizontal plane (HP, Table 2). The plane crossing the anatomic landmarks nasion and sella, and perpendicular to the horizontal plane, was defined as the midsagittal reference plane (MSP). Finally, the plane perpendicular to the horizontal plane and MSP and passing through left porion was defined as the coronal plane (CP). The Slicer software utilized a 3D patient coordinate system (R, A, S; right-left, anterior–posterior and superior-inferior, respectively) corresponding to the x, z, y Cartesian coordinate system; accordingly, the RAS system was used in the present study. For the current study, a specifically developed Slicer extension module “Align2FH_SagittalPlane” was used to align the horizontal plane along the x–z plane such that *y* = 0 (or *S* = 0) and the MSP along the y–z plane such that *x* = 0 (or *R* = 0). In addition, the porion was positioned on the x axis, such that *y* = *z* = 0 (or *S* = *A* = 0). Subsequently, the “Transform tool” allowed automatic orientation of the CBCT volume and the corresponding reconstructed model in 3D space based on the predefined reference planes (Fig. 1). A similar methodology was used for the optimal orientation of 3D models of the control subjects.

**Table 2:** Definitions of the landmarks and reference planes used in the study

S. No		Landmarks	Abbreviation	Definition	Reference Author, year
1	**Midline landmarks**	Anterior nasal spine	ANS	Tip of the anterior nasal spine of the palatal bone in the hard palate	Jung et al., 2009 [27]
2		Pt A	Pt A	The point of maximum concavity on the contour of the premaxilla below the ANS	Damstra et al., 2012 [30]
3		Upper incisor midpoint	UIM	Contact point between the upper central incisors	Jung et al., 2009 [27]
4		Lower incisor midpoint	LIM	Contact point between the lower central incisors	Jung et al., 2009 [27]
5		Pt B	Pt B	The point of maximum concavity at the midline on the alveolar process of the mandible	Leung et al., 2018 [28]
6		Pogonion	Pog	The most anterior point in the symphysis	Jung et al., 2009 [27]
7		Menton	Me	The most inferior point in the symphysis	Jung et al., 2009 [27]
8	**Bilateral landmarks**	Infraorbital foramen	IOF	The external opening of the infraorbital canal, on the anterior surface of the body of maxilla on the right and left sides	
9		Zygion	Zyg	Most anterior, lateral point on the zygomatic arch in the frontal view on the right and left sides	Ercan et al., 2013 [31]
10		Canine fossa	CF	A depression on the anterior surface of the maxilla below the infraorbital foramen and on the lateral side of the *canine* eminence on the right and left sides	
11		Pyriform aperture	PA	The most concave point on the pyriform aperture	
12		Lowest pyriform aperture	LPA	The lowermost point on the concavity of the pyriform aperture	
13		Maxillary tuberosity	MT	Point of maximum convexity on the maxillary alveolar ridge on the right and left sides	
14		Convex point on the zygoma	Cx Z	The most convex part of the zygomatic bone (malar) in the lateral view	
15		Upper canine	UC	The most prominent point on the buccal surface of the upper canine	Leung et al., 2018 [28]
16		Lower canine	LC	The most prominent point on the buccal surface of the lower canine	Leung et al., 2018 [28]
17		Upper 1st molar	UM1	Mesiobuccal cusp of the upper 1^st^ molar on the right and left sides	Leung et al., 2018 [28]
18		Lower 1st molar	LM1	Mesiobuccal cusp of the lower 1^st^ molar on the right and left sides	Leung et al., 2018 [28]
19		Mental foramen	MF	Anterior opening of the mandibular canal on the body of the mandible lateral to and above the *mental* tubercle on the right and left sides	Suzuki et al., 2015 [29]
20		Lateral chin points	CP	The most anterior point of the chin on the outline of the mandibular symphysis at the lower canine region on the right and left sides	Leung et al., 2018 [28]
21		Gonion lateralis	GoL	Most lateral point between the mandibular corpus and the ramus junction on the right and left sides	Nur et al., 2016 [4]
22		Gonion inferius	GoI	Most inferior point between the mandibular corpus and the ramus junction on the right and left sides	Nur et al., 2016 [4]
23		Gonion posterius	GoP	Most posterior point between the mandibular corpus and the ramus junction on the right and left sides	Nur et al., 2016 [4]
24		Antegonial notch	AGo	Deepest point of the concavity between the mandibular corpus and the ramus junction on the right and left sides	Nur et al., 2016 [4]
25		Condylar	Con	Most superior midpoint of the condylar head on the right and left sides	Nur et al., 2016 [4]
26		Coronoid	Crn	The most superior point of the right coronoid process on the right and left sides	Leung et al., 2018 [28]
27		Sigmoid notch	Sig	The depth of concavity at the right sigmoid notch on the right and left sides	Leung et al., 2018 [28]
28		Orbitale	Or	The most inferior point of the lower margin of the bony orbit on the right and left sides	Damstra et al., 2012 [30]
29		Porion	Por	The most superior point of the external auditory meatus on the right and left sides	Leung et al., 2018 [28]
30		Nasion	Na	Midpoint of the frontonasal suture	Nur et al., 2016 [4]
31		Sella	S	Center of the hypophyseal fossa	Nur et al., 2016 [4]
		**Reference Planes**			
1		Horizontal plane	HP	A plane passing through the bilateral orbitales and right porion	Nur et al., 2016 [4]
2		Midsagittal plane	MSP	A plane perpendicular to the HP and passing through the nasion and sella	Nur et al., 2016 [4]
3		Coronal plane	CP	A plane perpendicular to the HP and MSP and passing through the right porion	Nur et al., 2016 [4]

#### Registration of pretreatment and posttreatment CBCT volumes

**Fig. 2:**
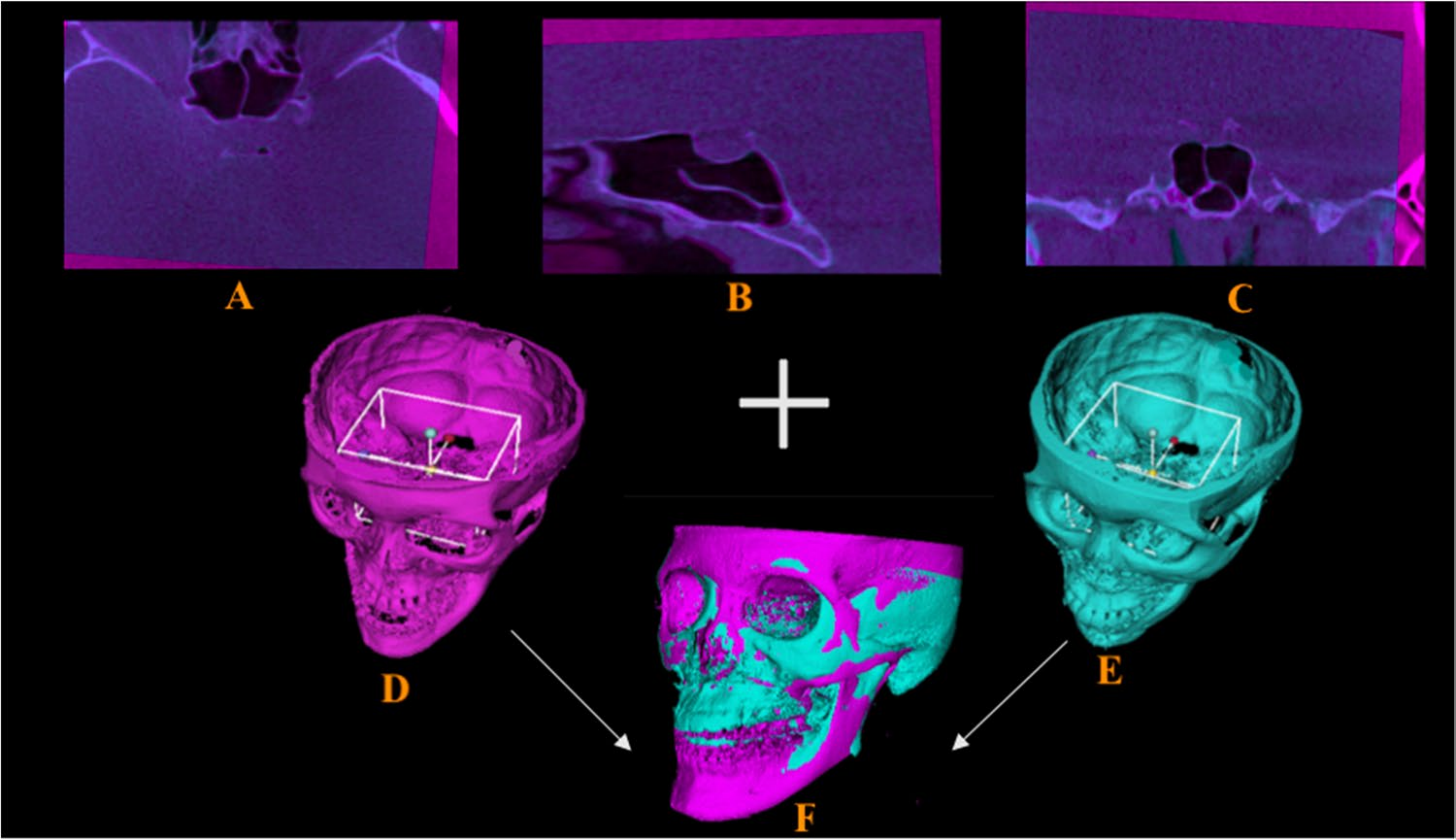
Representative images depicting the ROI for the registration of pre- and postoperative CBCT volumes based on the predefined stable structures of the cranium, where in pink signifies presurgery CBCT scan and blue signifies postsurgery CBCT scan. **A**, **B**, **C** ROI in axial, sagittal, and coronal views; **D**, **E** ROI in 3D reconstructed images (pre- and postsurgery respectively); **F** superimposed final 3D reconstructed image

For each patient, a semiautomated registration technique was utilized for the superimposition of preoperative CBCT with postoperative CBCT, which included initial manual rough alignment, followed by automatic fine alignment. The preliminary step in the superimposition of *T*_0_ and *T*_1_ 3D virtual models involved the selection of a region of interest (ROI) for both *T*_0_ and *T*_1_ CBCT volumes individually. This ROI was selected based on the predefined stable cranial structures not affected by the surgery. Next, the ROI was cropped from both volumes (*T*_0_ and *T*_1_) to specify the region for registration. Subsequently, the “Transforms tool” allowed superimposition of the cropped *T*_0_ and *T*_1_ volumes for initial manual alignment. Finally, fine alignment was automatically performed using the “General registration (BRAINS) tool,” thereby geometrically aligning the two volumes in the same 3D patient coordinate system (RAS) (Fig. 2).

### Assessment of asymmetry

#### Landmarks and measurements

**Fig. 3:**
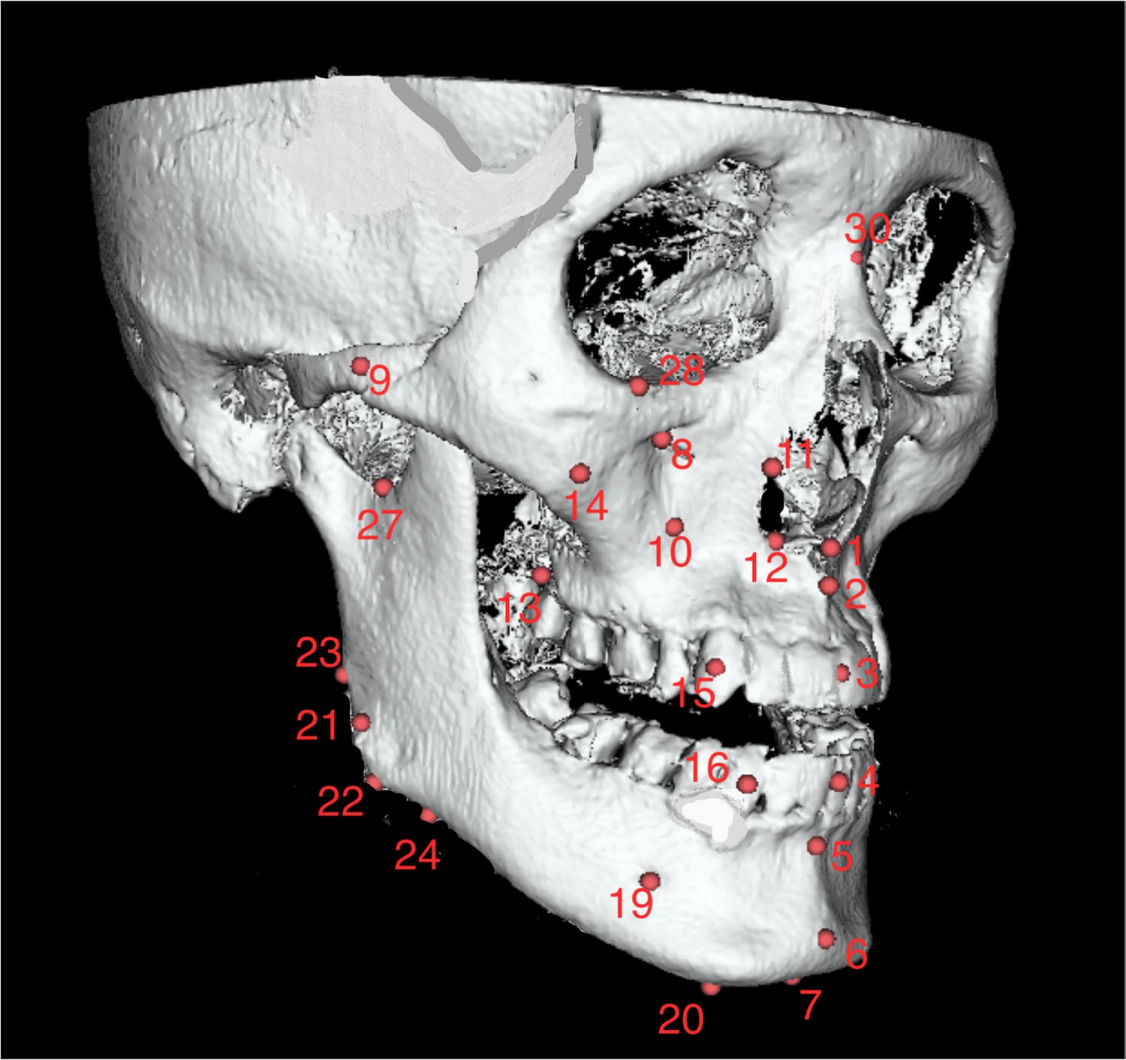
3D volume rendering of a skull showing various landmarks used in the study

After registration of the 3D images, 7 midline and 20 bilateral hard tissue landmarks [4, 27, 28], shown in Table 2, were identified on *T*_0_ (before surgery) scans, *T*_1_ (at least 6 months after surgery) scans, and scans of control patients. The digitized landmarks were manually placed on the 3D reconstructed model (Fig. 3) followed by verification of their location in all 3 planes. The left, posterior, and superior sides of the face were represented by negative coordinate values, and a positive value indicated the opposite sides.

#### Outcome measures

For the purpose of evaluating the maxillomandibular asymmetry, the distances of each landmark from the three reference planes were quantified as *d*_R_, *d*_A_, and *d*_S_ in millimeters (mm). The mean distance (*d*_R_, *d*_A_, and *d*_S_) of each landmark on the deviated side was compared with the nondeviated side within *T*_0_, *T*_1_, and controls to quantify the degree of baseline subclinical maxillomandibular asymmetry. Next, the mean differences of each landmark between the deviated and nondeviated sides were compared between *T*_0_ and controls, which indicated the site and severity of maxillomandibular asymmetry. Likewise, the effects of surgery on maxillomandibular asymmetry were assessed by comparing the mean differences of each landmark between *T*_0_ and *T*_1_. Finally, the mean differences of each landmark between *T*_1_ and controls were compared, which indicated the site and severity of any postoperative residual asymmetry. Residual asymmetry was defined as a measure of significant mean difference between *T*_1_-C, following significant mean differences between *T*_0_-*C* and *T*_0_ and *T*_1_.

#### Error study

All measurements were carried out by one investigator. Thirteen randomly selected CBCT images from each group (total 26) were remeasured in a 2-week interval and analyzed for intraexaminer reliability, and random error was calculated using the Dahlberg formula [29] for R, A, and S coordinates separately [30].

### Statistical analysis

Statistical analyses were performed using IBM SPSS Statistics for Mac, version 25.0 (IBM Corp., Armonk, N.Y., USA). The Shapiro–Wilk test was used to evaluate the normality of the data distribution. Preoperative and postoperative measured variables were compared using a paired *t* test. Likewise, the means of presurgery variables and postsurgery variables were compared with those of controls using an independent *t* test. Statistical interference of multiple comparisons was adjusted using Bonferroni correction (*p* < 0.05/number of tests, as statistically significant) to minimize the probability of falsely rejecting the null hypotheses, and a significance level of *p* < 0.003 (0.05/20) for intragroup and *p* < 0.002 (0.05/27) for intergroup differences was accepted as statistically significant.

## Results

From the initial pool of 27 orthognathic surgery cases, 5 patients without genioplasty and 1 patient who underwent one jaw surgery were excluded after screening to maintain the homogeneity of the subjects, resulting in a final sample of twenty-one patients, including 7 males and 14 females, with a mean age of 23.0 ± 3.4 years. In addition, the control group consisted of twenty-one age- and sex-matched adult patients (7 males, 14 females; mean age: 23.0 ± 3.3 years). The intraexaminer reliability for the measurements was excellent, with a mean intraclass correlation coefficient (ICC) of 0.95 (range: 0.90 to 0.99), and the method error ranged between 0.03 and 0.38 mm.

### Comparison between sides and groups in the right-left direction

A comparison of the mean distance and mean difference of each landmark between the deviated and nondeviated sides in the right-left (R-L) direction is summarized in Table 3. After Bonferroni correction, a cutoff value of *p* < 0.003 was considered statistically significant for the comparison between sides. Accordingly, a comparison of objective measurements between the deviated and nondeviated sides revealed that most of the bilateral landmarks in the midface and mandible were significantly deviated in the presurgical group (*T*_0_, Table 3). Even after surgery, a significant deviation was noticed at several landmarks in the mandible (LC, LM1, MF, LCP, GoL, and AGo) and at PA and UM1 in the midface (*T*_1_, Table 3). Interestingly, the chin peripheral region (mental foramen and lateral chin point) was found to be significantly deviated in the control sample (*C*, Table 3). Furthermore, following a comparison between the *T*_0_ and controls (Table 4), several sites at the mandible and midface were found to be affected by asymmetry. However, after Bonferroni adjustment (*p* < 0.002 for intergroup comparisons), asymmetry was more evident at the mandibular sites, specifically at the landmarks in the midline or close to the midline (LC, MF, and LCP). Postsurgically, a significant decrease in asymmetry characteristics with respect to the mandible and ANS was observed (*T*_0_ vs *T*_1_, Table 4). Adjustment of the significance level (Bonferroni-adjusted *p* < 0.002) revealed a substantial correction of mandibular midline landmarks (LIM, Pt B, Pog, and Me; *T*_0_ vs *T*_1_, Table 4) and the adjacent sites (LC, LM1, and LCP; *T*_0_ vs *T*_1_, Table 4). In fact, Me correction reached clinically normal (postsurgery soft tissue chin deviation < 3 mm) values (2.90 mm, *p* < 0.001) postsurgically (*T*_1_, Table 4). Likewise, postsurgical results for LIM and LC were comparable to those of controls (*p* = 0.096 and 0.245, respectively; *T*_1_ vs *C*, Table 4). Although a corrective change was also noticed at the mental foramen (from 13.13 to 7.65 mm) postsurgically, this change was not significant (*p* = 0.004; *T*_1_, Table 4). In addition, some degree of residual asymmetry was observed at Pt B, Pog, Me, LM1, MF, and LCP when compared with controls (*T*_1_ vs *C*, Table 4). However, after Bonferroni adjustment, the residual asymmetry was insignificant for the aforementioned landmarks except for the mental foramen. Indeed, compared with that of controls, the mental foramen showed some degree of residual asymmetry (*p* = 0.001).

**Table 3:** A comparison of measurements between deviated and nondeviated sides in the right-left (R) direction

Landmarks	**Comparison between sides**														
	Asymmetry group (*n* = 21)										Control group (*n* = 21)				
	**T0**					**T1**					**C**				
	**Deviated side**		**Nondeviated side**			**Deviated side**		**Nondeviated side**			**Deviated side**		**Nondeviated side**		
**Bilateral**	Mean	SD	Mean	SD	***p***	Mean	SD	Mean	SD	***p***	Mean	SD	Mean	SD	***p***
IOF	28.33	2.54	26.54	1.88	0.013*	28.07	1.91	26.95	2.92	0.150	26.54	2.19	26.18	1.84	0.564
Zyg	66.70	4.08	65.43	3.54	0.288	67.00	3.83	65.59	3.72	0.235	65.57	3.18	65.23	3.39	0.738
CF	25.68	3.26	22.71	2.45	0.002†	26.42	3.63	24.14	2.63	0.025*	24.09	2.23	23.75	2.71	0.661
PA	13.51	1.47	11.44	1.25	0.000†	13.75	1.14	11.59	2.60	0.001†	12.59	1.47	12.30	1.40	0.512
LPA	7.70	1.38	5.05	1.53	0.000†	7.23	1.88	5.59	2.02	0.010*	6.85	1.35	6.09	1.43	0.086
MT	31.71	3.12	27.96	2.24	0.000†	30.93	2.74	29.03	2.78	0.032*	31.14	2.12	30.56	2.60	0.429
CxZ	44.78	4.25	41.69	3.20	0.011*	44.47	3.82	43.53	3.29	0.403	43.86	3.05	43.76	3.69	0.925
UC	19.32	1.97	15.63	1.70	0.000†	19.76	1.73	17.89	10.20	0.412	18.18	2.25	17.00	2.19	0.094
LC	19.97	3.32	8.27	3.92	0.000†	16.48	1.82	12.01	1.91	0.000†	15.28	3.02	12.64	1.69	0.001†
UM1	27.70	2.42	23.23	1.71	0.000†	27.20	2.51	23.49	1.87	0.000†	24.76	2.78	23.54	2.40	0.136
LM1	28.37	3.32	18.75	4.27	0.000†	24.92	1.95	20.84	2.73	0.000†	21.69	1.84	19.67	1.87	0.001†
MF	30.31	4.36	17.30	5.05	0.000†	27.30	2.80	19.64	2.21	0.000†	24.78	2.17	21.43	1.49	0.000†
CP	21.94	4.90	9.13	3.92	0.000†	19.42	2.45	12.88	3.45	0.000†	15.55	2.38	12.20	2.36	0.000†
GoP	49.08	3.97	44.25	3.94	0.000†	49.26	4.03	46.66	5.70	0.095	46.99	3.78	44.71	3.88	0.061
GoI	49.64	3.93	42.80	4.68	0.000†	49.10	4.64	44.80	4.64	0.005*	46.49	3.80	43.26	3.39	0.006*
GoL	51.73	4.06	45.26	3.68	0.000†	51.73	3.64	47.10	4.70	0.001†	49.03	3.33	46.33	3.65	0.017*
AGo	46.66	4.51	38.43	4.67	0.000†	46.05	2.54	39.08	4.28	0.000†	43.00	3.29	39.88	3.34	0.004*
Crn	51.79	3.06	47.60	3.19	0.000†	48.85	2.96	49.24	5.73	0.784	48.84	3.25	48.62	3.45	0.836
Sig	51.81	3.16	48.41	2.81	0.001†	52.21	5.14	49.03	3.36	0.023*	49.57	2.09	48.79	2.78	0.308
Con	51.54	2.34	51.00	2.77	0.503	51.70	2.84	50.11	2.38	0.050	50.26	2.68	49.92	3.09	0.703

*T*_0_, presurgery; *T*_1_, postsurgery; *C*, control

Data are presented as means (mm) and SDs (mm)

^*^p < 0.05; Bonferroni-adjusted *p* value: †p < 0.003 (intragroup)

**Table 4:** A comparison of measurements between different groups in the right-left (R) direction

Landmarks	**Comparison between groups**																
	Asymmetry group										Control group						
	***T***_**0**_					***T***_**1**_					***C***				***T***_**0**_**-*C***	***T***_**0**_**-*T***_**1**_	***T***_**1**_**-*C***
	Mean	SD	**95% CI**			Mean	SD		**95% CI**		Mean	SD	**95% CI**		***p***	***p***	***p***
**Midline**			Lower		Upper				Lower	Upper			Lower	Upper			
ANS	1.58	0.83	0.25		1.19	1.19	0.86		0.00	0.78	0.86	0.65	− 0.15	0.80	0.003*	0.048*	0.175
Pt A	1.47	1.06	− 0.02		1.08	1.02	0.75		− 0.01	0.92	0.95	0.65	− 0.02	1.08	0.059	0.057	0.728
UIM	2.34	1.75	0.50		2.19	1.94	1.25		− 0.44	1.24	1.00	0.68	0.31	1.58	0.003*	0.328	0.005*
LIM	5.98	3.49	2.66		5.95	2.43	1.54		2.02	5.08	1.68	1.29	− 0.14	1.64	0.000‡	0.000‡	0.096
Pt B	6.44	3.91	3.39		6.95	2.58	1.78		2.44	5.27	1.27	0.94	0.42	2.20	0.000‡	0.000‡	0.006*
Pog	7.26	4.21	4.03		7.86	3.29	2.39		2.48	5.47	1.32	1.01	0.80	3.14	0.000‡	0.000‡	0.002*
Me	7.31	4.10	4.24		7.93	2.90	2.54	3.12		5.70	1.22	0.80	0.47	2.88	0.000‡	0.000‡	0.008*
**Bilateral**																	
IOF	2.48	1.69	− 0.60	1.38		2.21	1.74		− 0.24	0.78	2.09	1.47	− 0.88	1.13	0.431	0.284	0.807
Zyg	1.66	1.22	− 0.18	1.25		1.60	1.25		− 0.25	0.36	1.12	1.06	− 0.24	1.21	0.139	0.730	0.186
CF	3.28	2.72	− 0.48	2.42		3.75	2.27		− 1.41	0.49	2.31	1.85	0.14	2.73	0.185	0.324	0.031*
PA	2.46	1.59	− 0.16	1.71		2.96	2.48		− 1.65	0.64	1.68	1.39	0.03	2.54	0.100	0.368	0.046*
LPA	2.58	1.95	− 0.43	1.71		3.15	2.06		− 1.53	0.39	1.94	1.42	0.10	2.31	0.233	0.231	0.033*
MT	3.74	2.68	0.18	2.87		2.98	1.88		− 0.06	1.59	2.22	1.45	− 0.29	1.81	0.028*	0.068	0.150
CxZ	3.59	2.09	− 0.20	2.33		2.91	1.84		− 0.47	1.84	2.53	1.95	− 0.81	1.57	0.096	0.230	0.520
UC	3.82	3.06	− 0.04	3.07		5.01	4.48		− 3.39	1.00	2.31	1.73	0.58	4.83	0.056	0.270	0.016*
LC	11.7	7.11	4.82	11.67		4.57	3.03		4.06	10.20	3.45	3.08	− 0.79	3.02	0.000‡	0.000‡	0.245
UM1	4.57	3.15	0.78	4.00		3.49	2.59		− 0.35	2.52	2.18	1.82	− 0.09	2.71	0.005*	0.130	0.067
LM1	9.78	6.32	4.09	9.91		4.37	2.80		2.82	8.00	2.78	1.88	0.10	3.08	0.000‡	0.000‡	0.037*
MF	13.13	8.88	5.46	13.64		7.65	4.39		2.01	8.95	3.58	2.66	1.80	6.34	0.000‡	0.004*	0.001‡
CP	12.81	8.51	5.28	13.16		6.95	4.30		2.72	8.99	3.59	2.68	1.13	5.60	0.000‡	0.001‡	0.004*
GoP	5.16	3.54	− 0.12	3.74		7.51	10.17		− 7.11	2.42	3.35	2.57	− 0.47	8.79	0.065	0.316	0.077
GoI	7.09	5.03	0.51	5.65		6.02	3.80		− 1.80	3.94	4.01	2.93	− 0.11	4.13	0.021*	0.446	0.062
GoL	6.82	4.34	0.98	5.50		5.95	5.00		− 1.69	3.41	3.58	2.71	− 0.13	4.89	0.007*	0.488	0.065
AGo	8.90	6.35	1.56	7.74		4.36	7.23		− 0.70	4.03	4.25	2.93	0.66	5.30	0.005*	0.157	0.014*
Crn	4.38	3.22	1.37	4.37		7.03	13.24		− 8.96	3.67	1.51	1.07	− 0.35	11.37	0.000‡	0.393	0.072
Sig	3.70	2.29	0.50	2.85		5.10	11.06		− 6.16	3.36	2.03	1.36	− 1.85	7.99	0.007*	0.547	0.214
Con	1.66	1.31	− 0.55	1.00		1.97	1.66		− 0.84	0.23	1.44	1.17	− 0.37	1.43	0.567	0.243	0.240

*T*_0_, presurgery; *T*_1_, postsurgery; *C*, control

Data are presented as means (mm) and SDs (mm)

^*^p < 0.05; Bonferroni-adjusted *p* value; ‡*p* < 0.002 (intergroup)

### Comparison between sides and groups in the anteroposterior (A-P) and superoinferior (S-I) directions

The results of sidewise and groupwise comparisons of mean distances and mean differences in the A-P and S-I directions are illustrated in Tables 5, 6, 7, and 8. A Bonferroni-adjusted “*p*” value of < 0.003 was considered significant for intra- and intergroup comparisons, which revealed no significant deviation between deviated and nondeviated sides of the presurgery group in the anteroposterior (*T*_0_, Table 5) and superoinferior (*T*_0_, Table 6) directions. Likewise, there was no significant difference between the deviated and nondeviated sides of the postsurgery group (*T*_1_, Tables 5 and 6) and the control group (*C*, Tables 5 and 6). Regarding intergroup comparisons, A-P and S-I asymmetry was apparent at several sites of the mandible and midface before surgery compared with the corresponding sites in controls (*T*_0_-*C*, Tables 7 and 8). However, after Bonferroni adjustment, only LC (*p* = 0.001) was found to be asymmetric in the S-I direction (*T*_0_-*C*, Table 8). After correction, no apparent effect of surgery was noticed in the A-P and S-I directions (*T*_0_-*T*_1_, Tables 7 and 8). Moreover, postsurgical results were not comparable with control values in the A-P and S-I directions (*T*_1_-*C*, Tables 7 and 8). In addition, A-P and S-I asymmetry persisted at some of the landmarks of the mandible and midface in the postsurgery group compared with controls. Interestingly, this persistent asymmetry was insignificant after adjustment for the significance level, except for the sigmoid notch, which showed significant asymmetry post surgery (*p* = 0.001, *T*_1_-*C*, Table 8) in the S-I direction.

**Table 5:** A comparison of measurements between deviated and nondeviated sides in the anteroposterior (A) direction

Landmarks	**Comparison between sides**															
	Asymmetry group (*n* = 21)											Control group (*n* = 21)				
	***T***_**0**_					***T***_**1**_						***C***				
	**Deviated side**		**Nondeviated side**			**Deviated side**		**Nondeviated side**				**Deviated side**		**Nondeviated side**		
**Bilateral**	Mean	SD	Mean	SD	***p***	Mean	SD	Mean	SD	***p***	Mean		SD	Mean	SD	***p***
IOF	74.47	3.32	74.35	3.66	0.918	74.54	3.05	74.70	3.76	0.876	73.82		3.50	73.85	4.06	0.984
Zyg	37.98	2.83	38.31	4.03	0.757	37.71	3.24	37.68	4.14	0.985	35.56		3.77	34.53	4.44	0.424
CF	71.60	3.70	72.20	3.17	0.577	72.48	3.53	73.52	3.49	0.346	71.22		4.11	71.13	4.23	0.944
PA	80.28	3.62	80.76	3.49	0.667	81.03	3.89	80.82	3.22	0.848	81.07		3.83	80.69	4.23	0.766
LPA	78.82	4.28	78.79	4.25	0.982	77.12	17.62	80.64	3.95	0.378	79.37		3.73	79.20	4.00	0.888
MT	44.42	4.41	44.97	4.32	0.684	48.56	3.74	49.77	3.67	0.298	47.64		5.98	46.33	5.11	0.451
CxZ	69.98	2.80	70.43	3.13	0.630	70.23	2.74	69.85	3.17	0.673	68.17		3.73	67.96	4.16	0.863
UC	83.79	5.37	83.20	4.59	0.705	84.72	4.39	84.32	4.17	0.770	82.80		5.89	82.59	6.20	0.915
LC	87.57	5.56	88.02	5.60	0.795	82.86	4.96	82.44	4.90	0.784	80.89		6.96	80.78	6.97	0.958
UM1	65.41	4.62	64.94	4.36	0.739	67.20	5.17	67.19	4.73	0.995	64.59		5.14	64.42	5.31	0.919
LM1	68.16	5.27	69.99	4.82	0.248	65.82	5.71	65.14	4.96	0.683	64.46		5.60	64.52	5.66	0.973
MF	71.65	7.29	72.86	6.49	0.573	67.56	6.30	67.30	5.20	0.885	64.26		7.25	64.09	8.11	0.945
CP	74.72	8.95	75.54	8.86	0.768	70.94	6.86	70.35	6.69	0.776	66.83		8.93	66.26	9.17	0.837
GoP	12.61	4.42	14.84	3.96	0.092	12.27	5.07	12.29	4.77	0.991	8.84		3.95	9.00	3.85	0.895
GoI	22.26	5.57	25.95	4.76	0.027*	21.44	6.53	21.04	6.23	0.839	18.93		5.55	19.11	6.44	0.920
GoL	19.25	4.68	21.48	4.43	0.122	18.51	5.83	19.46	5.68	0.598	15.43		5.02	15.64	5.48	0.894
AGo	38.43	4.67	34.07	5.47	0.121	31.32	5.88	32.86	5.80	0.399	28.79		6.79	28.67	7.19	0.957
Crn	46.28	4.28	48.08	4.22	0.177	46.46	4.64	46.02	4.79	0.762	46.02		2.72	45.23	3.51	0.418
Sig	31.10	4.03	32.19	3.18	0.336	29.64	4.91	28.88	4.30	0.601	29.81		3.08	29.26	3.24	0.576
Con	12.62	3.25	11.78	3.20	0.407	13.84	3.06	12.40	3.54	0.165	14.34		2.44	13.89	3.06	0.599

*T*_0_, presurgery; *T*_1_, postsurgery; *C*, control

Data are presented as means (mm) and SDs (mm)

^*^p < 0.05; Bonferroni-adjusted p value: †*p* < 0.003 (intragroup)

**Table 6:** A comparison of measurements between deviated and nondeviated sides in the superoinferior (S) direction

Landmarks	**Comparison between sides**														
	Asymmetry group (*n* = 21)										Control group (*n* = 21)				
	***T***_**0**_					***T***_**1**_					***C***				
	**Deviated side**		**Nondeviated side**			**Deviated side**		**Nondeviated side**			**Deviated side**		**Nondeviated side**		
**Bilateral**	Mean	SD	Mean	SD	***p***	Mean	SD	Mean	SD	***p***	Mean	SD	Mean	SD	***p***
IOF	8.90	2.13	9.40	1.96	0.428	8.76	2.28	9.13	2.01	0.584	7.94	1.98	7.77	1.36	0.755
Zyg	1.37	1.33	1.62	1.31	0.547	1.73	1.31	1.47	1.25	0.516	1.30	0.82	0.99	0.77	0.219
CF	25.47	3.35	25.17	2.77	0.752	22.82	3.01	23.47	2.83	0.474	22.81	2.70	22.89	2.21	0.917
PA	17.10	2.91	16.27	2.79	0.352	15.28	2.94	15.54	2.60	0.767	14.24	2.49	14.03	2.47	0.791
LPA	25.98	2.72	25.13	3.13	0.358	25.07	2.87	25.45	3.35	0.695	24.72	2.37	24.57	2.44	0.842
MT	34.74	5.78	35.27	5.80	0.768	32.70	4.74	34.56	4.63	0.207	32.11	5.02	32.03	5.55	0.961
CxZ	15.93	1.68	16.05	2.08	0.839	15.93	1.82	16.08	2.42	0.821	16.47	1.52	16.97	1.99	0.365
UC	49.32	5.19	49.49	5.20	0.917	47.34	4.54	48.30	5.27	0.531	47.18	2.17	47.67	2.74	0.568
LC	60.08	7.85	60.79	7.31	0.762	60.06	4.68	60.85	5.46	0.619	63.85	3.50	64.12	3.71	0.813
UM1	49.92	5.33	50.21	4.85	0.854	47.63	3.96	48.30	4.55	0.613	47.88	3.21	48.29	3.01	0.676
LM1	54.07	7.03	55.16	6.45	0.605	52.46	4.41	53.63	4.39	0.395	57.06	3.32	57.53	3.59	0.661
MF	78.44	9.14	79.64	8.15	0.656	77.42	6.39	75.46	17.30	0.631	80.00	4.97	80.48	4.70	0.749
CP	95.74	10.57	95.88	10.39	0.964	94.23	7.94	94.60	7.89	0.883	96.81	5.15	96.99	4.83	0.907
GoP	45.15	5.61	46.71	5.28	0.359	44.58	5.58	46.20	4.33	0.300	50.23	5.34	51.37	5.31	0.493
GoI	64.89	7.79	66.72	7.92	0.454	63.88	7.84	64.95	6.44	0.634	68.21	6.17	70.18	6.49	0.320
GoL	56.18	7.66	55.30	14.01	0.802	55.36	6.99	56.80	6.72	0.499	59.37	5.59	61.77	5.95	0.186
AGo	69.71	8.12	71.46	6.80	0.454	65.30	6.74	68.09	5.99	0.164	71.57	6.31	73.27	5.74	0.367
Crn	8.71	4.89	11.82	5.16	0.052	6.27	3.35	8.56	5.34	0.104	13.15	4.84	13.45	5.05	0.846
Sig	19.58	3.71	21.00	3.26	0.197	17.10	3.07	19.44	3.81	0.035*	22.32	3.59	23.18	3.52	0.441
Con	2.74	1.86	2.88	1.69	0.802	2.60	1.87	1.97	1.37	0.221	2.37	1.95	2.29	1.61	0.888

*T*_0_, presurgery; *T*_1_, postsurgery; *C*, control

Data are presented as means (mm) and SDs (mm)

^*^p < 0.05; Bonferroni-adjusted p value: †*p* < 0.003 (intragroup)

**Table 7:** A comparison of measurements between different groups in the anteroposterior (A) direction

Landmarks	**Comparison between groups**														
	Asymmetry group								Control group						
	***T***_**0**_				***T***_**1**_				***C***				***T***_**0**_**-*C***	***T***_**0**_**-*T***_**1**_	***T***_**1**_**-*C***
**Bilateral**	Mean	SD	**95% CI**		Mean	SD	**95% CI**		Mean	SD	**95% CI**		***P***	***P***	***P***
			Lower	Upper			Lower	Upper			Lower	Upper			
IOF	1.56	0.96	− 0.38	1.12	1.53	1.02	− 0.39	0.46	1.19	1.40	− 0.42	1.11	0.320	0.878	0.373
Zyg	2.38	1.88	− 1.81	0.87	2.36	2.24	− 0.99	1.04	2.85	2.39	− 1.94	0.95	0.485	0.956	0.493
CF	1.78	1.34	− 0.89	0.77	1.78	1.81	− 0.92	0.91	1.84	1.33	− 1.05	0.93	0.883	0.996	0.906
PA	1.02	0.98	− 0.50	0.58	1.37	1.58	− 0.80	0.10	0.98	0.72	− 0.36	1.15	0.876	0.117	0.296
LPA	0.77	0.60	− 0.13	0.52	1.47	1.06	− 1.26	− 0.15	0.57	0.42	0.40	1.41	0.226	0.015*	0.216
MT	1.38	1.20	− 2.29	− 0.40	2.39	1.68	− 1.79	− 0.23	2.73	1.77	− 1.41	0.74	0.006*	0.014*	0.535
CxZ	1.92	1.29	− 1.33	0.47	1.75	1.28	− 0.34	0.68	2.35	1.57	− 1.50	0.30	0.340	0.489	0.183
UC	1.74	1.82	0.06	1.74	1.44	0.88	− 0.63	1.22	0.84	0.52	0.15	1.06	0.036*	0.516	0.010*
LC	1.72	2.08	− 0.27	1.76	0.84	0.90	− 0.10	1.88	0.98	0.84	− 0.70	0.41	0.147	0.077	0.599
UM1	1.47	1.13	− 0.01	1.13	1.60	1.14	− 1.42	0.27	0.91	0.61	0.39	1.87	0.056	0.173	0.005*
LM1	2.34	1.39	0.23	1.84	1.92	1.00	− 0.52	1.34	1.30	1.18	− 0.06	1.31	0.013*	0.365	0.072
MF	2.22	1.50	− 0.41	1.18	1.60	1.17	− 0.36	1.61	1.84	0.98	− 0.92	0.44	0.331	0.201	0.481
CP	1.80	1.52	− 0.21	1.46	1.68	1.22	− 0.71	0.95	1.17	1.12	− 0.23	1.24	0.141	0.769	0.174
GoP	2.93	1.78	− 0.80	1.37	2.50	1.96	− 0.97	1.83	2.65	1.67	− 1.29	0.99	0.598	0.526	0.793
GoI	4.38	1.82	− 1.08	2.01	4.19	3.26	− 1.30	1.68	2.56	1.95	− 1.64	1.92	0.003*	0.789	0.059
GoL	3.79	2.60	2.62	4.98	3.48	3.28	− 1.51	2.14	3.33	2.34	2.27	4.41	0.550	0.721	0.872
AGo	3.59	2.48	0.08	2.60	2.82	2.61	− 0.94	2.49	2.25	1.42	− 0.74	1.88	0.039*	0.358	0.388
Crn	3.01	2.12	− 0.94	1.57	2.55	2.57	− 0.76	1.68	2.70	1.90	− 1.56	1.27	0.617	0.443	0.835
Sig	3.39	2.22	− 0.28	2.22	3.41	2.76	− 1.35	1.32	2.42	1.74	− 0.46	2.43	0.123	0.982	0.174
Con	2.70	2.36	− 1.49	1.28	2.89	2.26	− 1.10	0.72	2.81	2.06	− 1.27	1.43	0.876	0.672	0.906

*T*_0_, presurgery; *T*_1_, postsurgery; *C*, control

Data are presented as means (mm) and SDs (mm)

^*^p < 0.05; Bonferroni-adjusted p value: †*p* < 0.003 (intergroup)

**Table 8:** A comparison of measurements between different groups in the superoinferior (S) direction

Landmarks	**Comparison between groups**														
	Asymmetry group								Control group						
	***T***_**0**_				***T***_**1**_				***C***				***T***_**0**_**-*C***	***T***_**0**_**-*T***_**1**_	***T***_**1**_**-*C***
**Bilateral**	Mean	SD	**95% CI**		Mean	SD	**95% CI**		Mean	SD	**95% CI**		***P***	***P***	***P***
			Lower	Upper			Lower	Upper			Lower	Upper			
IOF	1.12	0.92	− 0.72	0.47	1.12	0.76	− 0.34	0.33	1.24	0.97	− 0.67	0.42	0.674	0.994	0.651
Zyg	1.16	0.95	0.01	0.96	1.04	0.81	− 0.14	0.39	0.68	0.51	− 0.06	0.79	0.048*	0.346	0.094
CF	1.27	0.92	− 0.72	0.43	1.39	1.27	− 0.84	0.60	1.41	0.92	− 0.71	0.67	0.617	0.728	0.950
PA	1.60	1.00	− 0.23	0.93	1.54	0.87	1.14	− 0.56	0.70	0.83	− 0.25	0.82	0.225	0.823	0.289
LPA	0.89	0.86	0.09	0.91	0.94	0.78	− 0.43	0.32	0.39	0.34	0.17	0.93	0.019*	0.767	0.005*
MT	1.74	1.48	− 0.88	0.99	2.17	2.27	− 1.30	0.44	1.69	1.51	− 0.72	1.69	0.906	0.317	0.421
CxZ	0.83	0.96	− 0.61	0.42	1.35	0.90	− 1.00	− 0.04	0.93	0.67	− 0.07	0.92	0.714	0.034*	0.090
UC	1.08	1.03	− 0.14	1.01	1.39	1.30	− 1.13	0.52	0.65	0.80	0.06	1.41	0.138	0.449	0.034*
LC	1.72	1.44	0.52	1.91	1.29	1.16	− 0.43	1.18	0.51	0.54	0.20	1.36	0.001†	0.346	0.010*
UM1	1.60	1.14	0.06	1.33	2.01	1.59	− 1.22	0.40	0.90	0.87	0.31	1.91	0.033*	0.301	0.009*
LM1	1.75	1.67	0.21	1.81	1.51	1.42	− 0.70	1.20	0.75	0.72	0.06	1.46	0.017*	0.593	0.037*
MF	2.28	1.98	0.18	2.29	2.14	1.69	− 0.87	1.15	1.04	1.32	0.15	2.05	0.024*	0.776	0.024*
CP	1.34	1.17	0.01	1.16	1.21	0.74	− 0.35	0.60	0.75	0.58	0.04	0.88	0.046*	0.580	0.031*
GoP	2.59	2.17	− 1.68	0.84	3.45	2.76	− 2.06	0.34	3.01	1.86	− 1.03	1.91	0.506	0.150	0.547
GoI	2.97	2.22	− 0.96	1.66	4.15	3.32	− 2.53	0.17	2.62	1.96	− 0.18	3.23	0.597	0.084	0.079
GoL	2.71	2.31	− 1.89	1.03	4.41	3.69	− 3.23	− 0.18	3.14	2.36	− 0.66	3.20	0.555	0.030*	0.191
AGo	3.88	3.35	− 0.28	3.18	4.60	2.99	− 2.20	0.75	2.43	2.02	0.58	3.77	0.101	0.318	0.009*
Crn	3.61	2.92	− 0.15	2.93	3.28	2.71	− 0.93	1.59	2.22	1.92	− 0.40	2.53	0.077	0.594	0.151
Sig	2.24	1.54	0.14	1.79	2.97	1.95	− 1.80	0.34	1.28	1.06	0.71	2.68	0.023*	0.171	0.001†
Con	1.81	1.59	− 0.09	1.43	1.49	1.43	− 0.37	1.01	1.14	0.67	− 0.34	1.05	0.087	0.346	0.315

*T*_0_, presurgery; *T*_1_, postsurgery; *C*, control

Data are presented as means (mm) and SDs (mm)

^*^p < 0.05; Bonferroni-adjusted p value: †*p* < 0.003 (intergroup)

## Discussion

An accurate judgment and recognition of the site, degree, and severity of facial asymmetry is imperative for a better understanding of the etiology and for the accurate diagnosis and treatment planning for patients with dentofacial deformities [31]. A slight amount of facial asymmetry is innate to a normal face and is acceptable by an observer to be within the normal range [13]. Previous studies have shown that a menton deviation of 0–3 mm may be deemed normal; however, a deviation exceeding 3 mm may be defined as asymmetric [32–35]. Accordingly, the asymmetry group in the present study included patients with a menton deviation of ≥ 3.0 mm.

The construction of optimal facial planes based on landmarks that are minimally affected by facial asymmetry is a fundamental step for the clinical evaluation of facial asymmetry. In this regard, Kim et al. [36] suggested that the landmark-based reference plane was compatible with reference planes from Procrustes analysis. Therefore, for the present study, we defined reference planes on the basis of the landmark-based technique. Severt and Proffit showed that the upper face was comparatively stable (5%) to asymmetrical changes when compared against the midface (36%) and lower face (74%) [7]. Considering this fact, a plane passing through three landmarks from the upper face, viz., bilateral orbitale, and the right porion was selected to define the horizontal reference plane. In addition, previous studies have shown that the cranial base is impervious to facial asymmetry, and its morphological characteristics are similar in symmetric and asymmetric faces [37]. For the same reason, the sella and nasion, which are stable cranial base landmarks and are unaffected by asymmetry, were chosen for the construction of the midsagittal reference plane perpendicular to the horizontal plane [4, 38]. The coronal plane was then automatically adjusted to align perpendicular to the horizontal and midsagittal planes by Slicer software.

Fundamentally, there are three different ways to superimpose CBCT volumes: landmark-based, surface-based, and voxel-based superimposition. It is essential for a good superimposition method to precisely register and assist in understanding the changes resulting from growth and/or treatment in relation to the reference structure. The landmark-based method is considered to be imprecise owing to the difficulty in identifying landmarks on cephalograms. Moreover, lack of depth, magnification variance, and disparities in the head orientation make it a complex superimposition procedure [39, 40]. A surface-based method, on the other hand, utilizes a high-quality surface of the 3D structure for precise superimposition. A few other studies have also utilized Procrustes analysis for the superimposition of 3D imaging; however, the results showed errors of approximately 2 mm for some anatomical landmarks [41, 42]. Cevidanes et al. [39, 43] presented a voxel-based registration based on matching the grayscale values of the voxels in the area of reference for CBCT volume superimposition. It is a completely automated and observer-independent superimposition that was central for the present study to minimize observer-related errors [44, 45]. On account of its precision, the voxel-based method has been applied to evaluate postoperative changes in orthognathic surgery patients. Studies comparing surface-based and voxel-based methods have shown no statistically significant differences between the two methods when analyzing skeletal changes, and their accuracy has been validated; however, the voxel-based technique has been associated with less variability [46, 47]. In addition, all the contents of the selected volume are utilized for voxel-based registration, thereby theoretically increasing the accuracy of the technique [47].

Where most of the previous studies have focused on the horizontal (right-left) component of the asymmetry, which can be clinically appreciated, our study analyzed asymmetry in all three dimensions (R, A, and S). From the patient’s aesthetic perception point of view, although the right-left (R) asymmetry in the “R” coordinate was more crucial since it is easily detectable by the patient when looking into the mirror from the frontal view or during social interactions, we analyzed the asymmetry in “A” and “S” coordinates as well for the precise estimation of site, severity, and posttreatment outcomes. The present study provides deeper insights into the site, severity, and outcome measures by analyzing different regions of the face potentially affected by orthognathic surgery. Based on the comparative evaluation of various landmarks between asymmetric patients before surgery and the controls, we noticed that asymmetry was more severe in the mandibular region than in the midface. Consistent with the intragroup results, several mandibular landmarks were found to be asymmetric before Bonferroni adjustment in the intergroup comparisons. Even after adjustment of the significance level, asymmetry was found to be more severe at several mandibular sites, specifically at the mandibular midline (lower incisal midline, point B, pogonion, and menton; Table 4) and chin peripheral region (lower canine, mental foramen and lateral chin point; Table 4), which was consistent with the findings of previous studies [11, 17, 48, 49]. These findings are indicative of the fact that the mandibular midline and chin peripheral region contribute significantly to the overall facial asymmetry characteristics. A reasonable explanation for this finding could be sustained mandibular growth periods and rigid attachment of the maxilla to the stable synchondrosis region at the cranial base [31].

In the current analysis, we also analyzed asymmetry in A and S coordinates, i.e., in the anteroposterior (A-P) and superoinferior (S-I) directions. Although A-P and S-I asymmetry was evident at several mandibular and midfacial landmarks, nevertheless, following Bonferroni correction, only the lower canine showed significant asymmetry in the S-I direction. A-P and S-I asymmetry in dental landmarks such as the upper canine, lower canine, upper first molar, and lower first molar could be attributed to dental malocclusion such as tipping or supra-eruption/impaction; however, asymmetry of skeletal landmarks including the maxillary tuberosity, gonion inferius, and antegonion in the A-P direction and the zygion, lower pyriform aperture, mental foramen, lateral chin point, and sigmoid notch in the S-I direction, as seen in the present analysis, confirmed that A-P and vertical components of asymmetry exist. The significance of analyzing asymmetry in the A-P and S-I directions lies in the fact that although asymmetry is appreciated in the right-left direction, the A-P and S-I components remain unnoticed. Landmarks that seem symmetric in one dimension may not be symmetric in other dimensions. For instance, in the present study, zygion and lower pyriform apertures were symmetric in the R-L direction but revealed asymmetries in the vertical direction.

Well-planned orthognathic surgery can help achieve desired aesthetic results, as observed in the present study, wherein a significant correction of asymmetry characteristics with regard to the mandible in the R-L direction was observed, which was in accordance with previous studies [20]. Notably, postsurgical symmetry for the ANS, menton, lower incisor midline, and lower canine was equivalent to that in controls. Since chin deviation is the most apparent in facial asymmetry patients; correction of the chin was vital for postsurgical aesthetic perception [24, 50]. Accordingly, the results of the present analysis showed a significant improvement in the chin region (Pt B, pogonion, menton, and lateral chin point) in the R-L direction (Table 4). Additionally, it was noticed that landmarks that seem symmetric in one dimension after correction may not be symmetric in other dimensions. For instance, in the present study, the lower canine, lower first molar, and lateral chin point showed asymmetry in the R-L direction. After correction, considerable reduction in R-L asymmetry characteristics was noticed at the aforementioned landmarks; however, despite successful surgery, the vertical component of asymmetry persisted, thereby suggesting the necessity of three-dimensional presurgical planning for aesthetic postoperative outcomes. It is also worth noting that the degree of postoperative asymmetry was not related to the preoperative deviation.

Very few studies have reported residual asymmetry [51, 52]; moreover, to the best of our knowledge, there is no study comparing facial asymmetry-associated orthognathic surgery outcomes with the corresponding characteristics in the control population. An assessment of postoperative aesthetic outcomes revealed that although significant improvement was noticed, postsurgical outcomes were not comparable with controls, as mild asymmetry persisted in some regions (Pt B, pogonion, menton, lower molar, mental foramen, and lateral chin point), thereby suggesting the presence of “residual asymmetry” even after surgery (Table 4). These results were in agreement with the findings of Lin et al. [51], wherein significant residual asymmetry was reported at the symphysis-parasymphysis and mandibular body regions, which correspond to Pt B, pogonion, and menton, and lower molar, mental foramen, and lateral chin point sites, respectively. Although only the mental foramen showed substantial residual asymmetry after the adjustment of the significance level (Table 4), residual asymmetry seen at other sites (Pt B, pogonion, menton, lower first molar, and lateral chin point) indicates the need for secondary correction and cannot be underestimated if symmetric facial features are desired. In addition, some degree of asymmetry was also obvious at other sites post surgery, such as the upper incisal midline and antegonion in the R-L direction (Table 4); the upper canine in the A-P direction; and the lowermost point of the pyriform aperture, lower canine, upper first molar, lower first molar, mental foramen, lateral chin point, and sigmoid notch in the S-I direction (Table 8); nevertheless, the asymmetry observed was not true residual asymmetry per se (*T*_0_-*T*_1_ insignificant, while *T*_0_-*C*, and *T*_1_-*C* significant, respectively). This persisting asymmetry can be attributed to inadequate surgical correction. Since the residual asymmetry noticed in the present study was predominantly in the transverse direction (R-L), outer bone cortex grinding [53] could be performed as an adjunctive surgical modality for the correction of the residual asymmetry in a single surgery, thereby decreasing the need for secondary surgery.

Orthognathic surgery involves pitch, yaw, and roll of the osteotomized segments, which alter the initial position of the landmarks with respect to reference planes to achieve the desired position of the segments postsurgically [54]. Therefore, as a repercussion of surgery, the landmarks that were symmetric before surgery may appear asymmetric after surgery, giving a notion of induced asymmetry. Interestingly, in the present study, a similar phenomenon was noticed at several sites, namely, the canine fossa, pyriform aperture, lowermost point of the pyriform aperture, and upper canine in the R-L direction (Table 4); upper first molar in the A-P direction (Table 7); and upper canine and antegonion in the S-I direction (Table 8). Although this asymmetry is minimal, from an aesthetic point of view, all these components (R, A, and S) need to be addressed during the presurgical planning phase to achieve a nearly symmetric face, since surgery planned based on one dimension may influence postsurgical outcomes in other dimensions.

Regardless of the comprehensive analysis, some limitations should be considered for this study. First, the current study was a retrospective study; nevertheless, by selecting consecutive patients, this limitation was kept minimal. Second, the small sample size of this study limits the generalization of the conclusions. Increasing the number of patients may be required to delineate the results. Finally, although the landmark-based method for reference plane estimation has been shown to be comparable with semiautomatic and automatic techniques [36], errors related to the manual digitization of landmarks may exist, since digitization depends upon the ability of the observer to identify them precisely. However, the intraobserver reliability was excellent for the landmarks used in the current study. Furthermore, it would be interesting to develop presurgical diagnostic aids for the precise predictability of A-P and S-I components of residual asymmetry, thereby contributing to modifications in current surgical approaches for the accomplishment of desired postsurgical aesthetic outcomes. In addition, future studies are required to analyze the treatment outcomes with regard to soft tissues after bimaxillary surgery in patients with asymmetry.

## Conclusions

The present analysis emphasizes the importance of three-dimensional presurgical evaluation of and treatment planning for facial asymmetry. Asymmetric mandibular midline landmarks and chin peripheral regions contribute significantly to the overall facial asymmetry. In this study, orthognathic surgery resulted in significant correction of maxillomandibular asymmetry with clinically apparent correction in the mandible, especially at the menton. However, the mental foramen showed significant residual asymmetry after surgery. In addition, mild residual asymmetry also persisted at Pt B, the pogonion, the menton, the lower first molar, and the lateral chin point, even after surgery. Postsurgical asymmetry resulting from inadequate surgical correction or as a repercussion of surgery might be misjudged as residual asymmetry and should be evaluated with caution.

## Data Availability

The datasets used and/or analyzed during the current study are available from the corresponding author on reasonable request.
